# 3-Chloro-6-{4-[3-(4-chloro­phen­oxy)prop­yl]piperazin-1-yl}pyridazine

**DOI:** 10.1107/S1600536810005337

**Published:** 2010-02-27

**Authors:** Hongliang Wang, Junhai Xiao, Xian Zhang, Tiemin Sun, Song Li

**Affiliations:** a School of Pharmaceutical Engineering, Shenyang Pharmaceutical University, Shenyang 110016, People’s Republic of China; b Beijing Institute of Pharmacology and Toxicology, Beijing 100850, People’s Republic of China

## Abstract

In the title compound, C_17_H_20_Cl_2_N_4_O, the piperazine ring adopts a chair conformation and the dihedral angle between the pyridazine ring and the benzene ring is 36.3 (1)°. In the crystal, weak C—H⋯O and C—H⋯(N,N) inter­actions help to establish the packing, which also features short inter­molecular Cl⋯Cl contacts [3.331 (2) Å].

## Related literature

For the biological properties of 3-(piperazin-1-yl)pyridazine derivatives, see: Monge *et al.* (1991 ▶); Tucker *et al.* (1998 ▶). For the synthesis, see: Fan *et al.* (2009 ▶). 
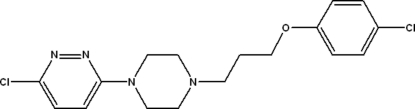


## Experimental

### 

#### Crystal data

C_17_H_20_Cl_2_N_4_O
*M* *_r_* = 367.27
Monoclinic, 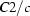

*a* = 39.774 (18) Å
*b* = 5.757 (3) Å
*c* = 14.924 (7) Å
β = 93.107 (9)°
*V* = 3412 (3) Å^3^
*Z* = 8
Mo *K*α radiation
μ = 0.39 mm^−1^
*T* = 113 K
0.20 × 0.18 × 0.08 mm

#### Data collection

Rigaku Saturn CCD area-detector diffractometer
Absorption correction: multi-scan (*CrystalClear*; Rigaku/MSC, 2005 ▶) *T*_min_ = 0.926, *T*_max_ = 0.969
11904 measured reflections
2996 independent reflections
2030 reflections with *I* > 2σ(*I*)
*R*_int_ = 0.061

#### Refinement

*R*[*F* ^2^ > 2σ(*F* ^2^)] = 0.050
*wR*(*F* ^2^) = 0.159
*S* = 1.09
2996 reflections
217 parameters
H-atom parameters constrained
Δρ_max_ = 0.30 e Å^−3^
Δρ_min_ = −0.33 e Å^−3^

### 

Data collection: *CrystalClear* (Rigaku/MSC, 2005 ▶); cell refinement: *CrystalClear*; data reduction: *CrystalClear*; program(s) used to solve structure: *SHELXS97* (Sheldrick, 2008 ▶); program(s) used to refine structure: *SHELXL97* (Sheldrick, 2008 ▶); molecular graphics: *SHELXTL* (Sheldrick, 2008 ▶); software used to prepare material for publication: *SHELXL97*.

## Supplementary Material

Crystal structure: contains datablocks I, global. DOI: 10.1107/S1600536810005337/hb5330sup1.cif

Structure factors: contains datablocks I. DOI: 10.1107/S1600536810005337/hb5330Isup2.hkl

Additional supplementary materials:  crystallographic information; 3D view; checkCIF report

## Figures and Tables

**Table 1:** Hydrogen-bond geometry (Å, °)

*D*—H⋯*A*	*D*—H	H⋯*A*	*D*⋯*A*	*D*—H⋯*A*
C3—H3⋯N1^i^	0.95	2.53	3.247 (6)	133
C3—H3⋯N2^i^	0.95	2.50	3.427 (6)	164
C13—H13⋯O1^ii^	0.95	2.60	3.529 (5)	168

Symmetry codes: (i) 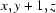; (ii) 
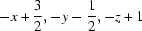
.

**Fig. 1.**
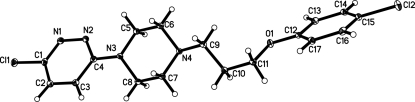
The molecular structure of (I) with displacement ellipsoids drawn at the 50% probability level.

**Fig. 2.**
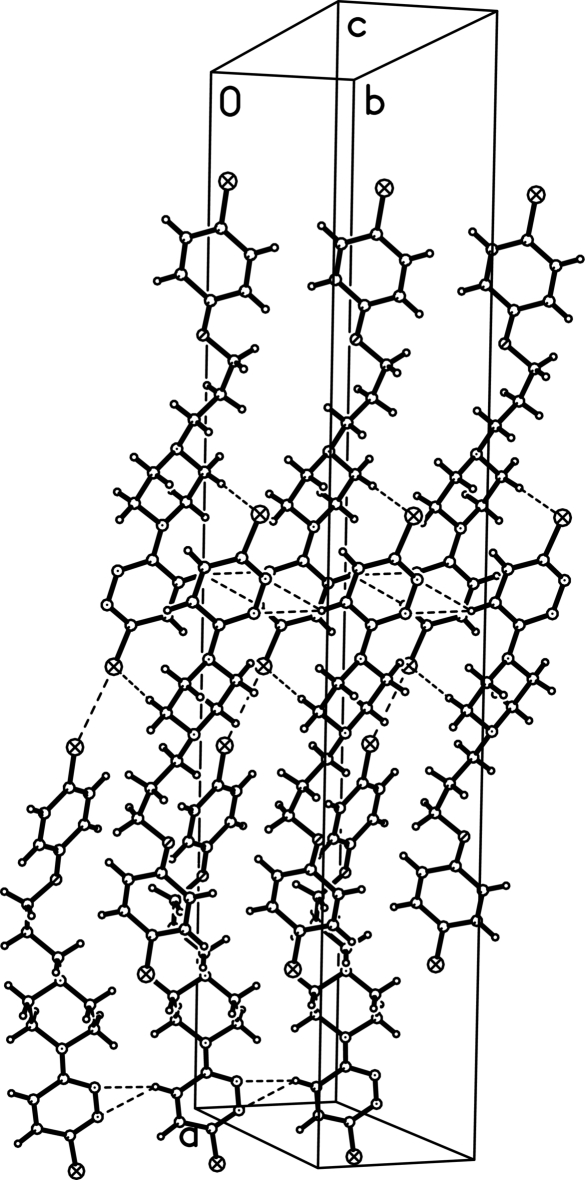
The crystal packing of (I) with Cl···Cl, C—H···N and C—H···O interactions shown as dashed lines.

## supplementary crystallographic information

### Comment

Pyridazine derivatives are important aromatic heterocycle compounds in the field of medicinal chemistry: for example, 3-(piperazin-1-yl)pyridazine derivatives are reported to possess anti-inotropic, anti-blood platelet aggregation (Monge *et al.*,  1991), anti-bacterial (Tucker *et al.*, 1998) and anti-viral activities (Fan *et al.*, 2009).

The diagram of the title compound is shown in Fig.1. The bond lengths and angles are generally within normal ranges. The piperazine ring in the molecule adopts chair conformation. The dihedral angle between the pyridazine ring and the benzene ring is 36.3 (1)°.

In the crystal structure, the molecules are linked by intermolecular Cl2···Cl1 (symmetry code: x, 1+y, z), C7—H7A···Cl1 (symmetry code: -1/2+x, -1/2-y, 1/2+z) and N1···H3···N2 (symmetry code: 2-x, -y, 1-z) interactions (Fig. 2).

### Experimental

Diethyl azodicarboxylate (0.002 mol) was added in small portions to a stirred solution of 3-(4-(6-chloropyridazin-3-yl)piperazin-1-yl)pro-1-ol (0.002 mol), 4-chlorophenol (0.002 mol) and triphenylphosphine (0.002 mol) in anhydrous THF (10 ml). The mixture was stirred for 24 h at room temperature (Shi-Yong Fan *et al.*, 2009). After removal of the THF under reduced pressure, the residue was purified by column chromatography (petroleum ether/acetone, 2:1, *v*/*v*) to afford the title compound as a colourless solid. Colourless prisms of (I) were prepared by slow evaporation of a solution of the title compound in ethanol at room temperature.

### Refinement

The C—H H atoms were placed in ideal positions and were refined using as riding model. With C—H=0.95 Å (aromatic), 0.99 Å (methylene) and *U*_iso_(H)=1.2*U*_eq_(C).

### Crystal data

**Table:**

C_17_H_20_Cl_2_N_4_O	*F*(000) = 1536
*M**_r_* = 367.27	*D*_x_ = 1.430 Mg m^−^^3^
Monoclinic, *C*2/*c*	Mo *K*α radiation, λ = 0.71073 Å
*a* = 39.774 (18) Å	Cell parameters from 5597 reflections
*b* = 5.757 (3) Å	θ = 2.1–28.0°
*c* = 14.924 (7) Å	µ = 0.39 mm^−^^1^
β = 93.107 (9)°	*T* = 113 K
*V* = 3412 (3) Å^3^	Prism, colourless
*Z* = 8	0.20 × 0.18 × 0.08 mm

### Data collection

**Table:**

Rigaku Saturn CCD area-detector diffractometer	2996 independent reflections
Radiation source: rotating anode	2030 reflections with *I* > 2σ(*I*)
multilayer	*R*_int_ = 0.061
Detector resolution: 14.63 pixels mm^-1^	θ_max_ = 25.0°, θ_min_ = 2.1°
ω and φ scans	*h* = −46→46
Absorption correction: multi-scan (*CrystalClear*; Rigaku/MSC, 2005)	*k* = −6→6
*T*_min_ = 0.926, *T*_max_ = 0.969	*l* = −15→17
11904 measured reflections	

### Refinement

**Table:**

Refinement on *F*^2^	Primary atom site location: structure-invariant direct methods
Least-squares matrix: full	Secondary atom site location: difference Fourier map
*R*[*F*^2^ > 2σ(*F*^2^)] = 0.050	Hydrogen site location: inferred from neighbouring sites
*wR*(*F*^2^) = 0.159	H-atom parameters constrained
*S* = 1.09	*w* = 1/[σ^2^(*F*_o_^2^) + (0.0834*P*)^2^] where *P* = (*F*_o_^2^ + 2*F*_c_^2^)/3
2996 reflections	(Δ/σ)_max_ = 0.003
217 parameters	Δρ_max_ = 0.30 e Å^−^^3^
0 restraints	Δρ_min_ = −0.33 e Å^−^^3^

### Special details

**Table:**

Geometry. All esds (except the esd in the dihedral angle between two l.s. planes) are estimated using the full covariance matrix. The cell esds are taken into account individually in the estimation of esds in distances, angles and torsion angles; correlations between esds in cell parameters are only used when they are defined by crystal symmetry. An approximate (isotropic) treatment of cell esds is used for estimating esds involving l.s. planes.
Refinement. Refinement of *F*^2^ against ALL reflections. The weighted *R*-factor wR and goodness of fit *S* are based on *F*^2^, conventional *R*-factors *R* are based on *F*, with *F* set to zero for negative *F*^2^. The threshold expression of *F*^2^ > σ(*F*^2^) is used only for calculating *R*-factors(gt) etc. and is not relevant to the choice of reflections for refinement. *R*-factors based on *F*^2^ are statistically about twice as large as those based on *F*, and *R*- factors based on ALL data will be even larger.

### Fractional atomic coordinates and isotropic or equivalent isotropic displacement parameters (Å^2^)

**Table:**

	*x*	*y*	*z*	*U*_iso_*/*U*_eq_	
Cl1	1.06826 (2)	−0.31802 (19)	0.30198 (6)	0.0238 (3)	
Cl2	0.64299 (2)	0.03379 (19)	0.75503 (6)	0.0252 (3)	
O1	0.77076 (5)	0.0299 (4)	0.57543 (16)	0.0172 (6)	
N1	1.01144 (7)	−0.4149 (6)	0.37004 (19)	0.0191 (8)	
N2	0.98066 (6)	−0.3697 (6)	0.40118 (19)	0.0177 (7)	
N3	0.93922 (6)	−0.1129 (5)	0.44101 (19)	0.0159 (7)	
N4	0.87083 (6)	−0.0635 (5)	0.49145 (19)	0.0149 (7)	
C1	1.02999 (8)	−0.2401 (7)	0.3457 (2)	0.0177 (9)	
C2	1.02128 (8)	−0.0067 (7)	0.3520 (2)	0.0182 (9)	
H2	1.0360	0.1133	0.3349	0.022*	
C3	0.99070 (8)	0.0412 (7)	0.3837 (2)	0.0182 (8)	
H3	0.9832	0.1967	0.3898	0.022*	
C4	0.97035 (8)	−0.1495 (7)	0.4072 (2)	0.0154 (8)	
C5	0.92000 (8)	−0.3152 (7)	0.4675 (2)	0.0180 (8)	
H5A	0.9353	−0.4301	0.4975	0.022*	
H5B	0.9090	−0.3892	0.4137	0.022*	
C6	0.89344 (8)	−0.2413 (7)	0.5311 (2)	0.0179 (9)	
H6A	0.8800	−0.3786	0.5471	0.021*	
H6B	0.9047	−0.1797	0.5869	0.021*	
C7	0.89091 (8)	0.1374 (7)	0.4665 (2)	0.0156 (8)	
H7A	0.9021	0.2057	0.5212	0.019*	
H7B	0.8759	0.2569	0.4384	0.019*	
C8	0.91722 (8)	0.0697 (7)	0.4018 (2)	0.0164 (8)	
H8A	0.9060	0.0130	0.3451	0.020*	
H8B	0.9309	0.2074	0.3879	0.020*	
C9	0.84595 (8)	−0.0032 (7)	0.5562 (2)	0.0169 (8)	
H9A	0.8579	0.0471	0.6128	0.020*	
H9B	0.8328	−0.1438	0.5694	0.020*	
C10	0.82186 (8)	0.1877 (7)	0.5241 (2)	0.0169 (8)	
H10A	0.8346	0.3345	0.5193	0.020*	
H10B	0.8123	0.1478	0.4635	0.020*	
C11	0.79342 (8)	0.2255 (7)	0.5855 (2)	0.0158 (8)	
H11A	0.8024	0.2378	0.6485	0.019*	
H11B	0.7814	0.3713	0.5693	0.019*	
C12	0.74176 (8)	0.0403 (7)	0.6207 (2)	0.0146 (8)	
C13	0.71859 (8)	−0.1366 (7)	0.6022 (2)	0.0171 (8)	
H13	0.7236	−0.2563	0.5612	0.020*	
C14	0.68822 (8)	−0.1390 (7)	0.6433 (2)	0.0163 (8)	
H14	0.6723	−0.2587	0.6301	0.020*	
C15	0.68135 (8)	0.0346 (7)	0.7035 (2)	0.0169 (8)	
C16	0.70436 (8)	0.2093 (7)	0.7240 (2)	0.0181 (8)	
H16	0.6995	0.3259	0.7664	0.022*	
C17	0.73474 (8)	0.2130 (7)	0.6819 (2)	0.0167 (8)	
H17	0.7506	0.3331	0.6951	0.020*	

### Atomic displacement parameters (Å^2^)

**Table:**

	*U*^11^	*U*^22^	*U*^33^	*U*^12^	*U*^13^	*U*^23^
Cl1	0.0167 (5)	0.0310 (7)	0.0242 (5)	0.0050 (4)	0.0056 (4)	0.0013 (4)
Cl2	0.0177 (5)	0.0342 (7)	0.0246 (5)	−0.0029 (4)	0.0080 (4)	−0.0021 (4)
O1	0.0139 (12)	0.0165 (16)	0.0217 (14)	−0.0030 (11)	0.0047 (10)	−0.0055 (12)
N1	0.0185 (15)	0.018 (2)	0.0211 (17)	0.0044 (13)	0.0046 (13)	0.0023 (14)
N2	0.0160 (15)	0.015 (2)	0.0222 (17)	0.0029 (13)	0.0045 (12)	0.0017 (14)
N3	0.0141 (14)	0.0119 (19)	0.0220 (17)	0.0007 (12)	0.0046 (12)	0.0033 (13)
N4	0.0135 (14)	0.0108 (19)	0.0207 (16)	0.0004 (12)	0.0046 (12)	0.0012 (13)
C1	0.0127 (16)	0.024 (3)	0.0170 (18)	0.0020 (16)	0.0017 (14)	−0.0004 (17)
C2	0.0168 (18)	0.017 (3)	0.021 (2)	−0.0021 (16)	0.0000 (15)	0.0007 (17)
C3	0.0179 (18)	0.014 (2)	0.022 (2)	0.0014 (15)	0.0007 (15)	0.0009 (17)
C4	0.0165 (17)	0.013 (2)	0.0164 (19)	0.0002 (15)	−0.0012 (14)	−0.0008 (16)
C5	0.0167 (17)	0.012 (2)	0.026 (2)	0.0007 (16)	0.0029 (15)	0.0022 (17)
C6	0.0166 (17)	0.013 (2)	0.024 (2)	−0.0004 (15)	0.0030 (14)	0.0042 (16)
C7	0.0166 (17)	0.011 (2)	0.020 (2)	0.0008 (15)	0.0024 (14)	0.0008 (16)
C8	0.0179 (17)	0.015 (2)	0.0172 (19)	0.0014 (15)	0.0034 (14)	0.0037 (16)
C9	0.0165 (17)	0.017 (2)	0.0170 (19)	−0.0013 (16)	0.0017 (14)	0.0026 (16)
C10	0.0167 (17)	0.015 (2)	0.0197 (19)	−0.0008 (15)	0.0038 (14)	0.0008 (16)
C11	0.0158 (17)	0.012 (2)	0.0197 (19)	−0.0019 (15)	0.0014 (14)	0.0010 (16)
C12	0.0130 (17)	0.016 (2)	0.0153 (18)	0.0018 (15)	0.0008 (13)	0.0033 (16)
C13	0.0194 (18)	0.014 (2)	0.0174 (19)	0.0016 (16)	0.0000 (14)	−0.0024 (16)
C14	0.0176 (17)	0.015 (2)	0.0166 (19)	−0.0045 (15)	−0.0015 (14)	0.0020 (16)
C15	0.0134 (17)	0.021 (2)	0.0166 (19)	0.0017 (16)	0.0036 (14)	0.0041 (16)
C16	0.0205 (18)	0.017 (2)	0.0164 (19)	0.0034 (16)	0.0002 (14)	−0.0034 (16)
C17	0.0157 (17)	0.018 (2)	0.0164 (19)	−0.0034 (15)	−0.0006 (14)	0.0004 (16)

### Geometric parameters (Å, °)

**Table:**

Cl1—C1	1.747 (3)	C7—C8	1.513 (4)
Cl2—C15	1.745 (3)	C7—H7A	0.9900
O1—C12	1.369 (4)	C7—H7B	0.9900
O1—C11	1.445 (4)	C8—H8A	0.9900
N1—C1	1.311 (5)	C8—H8B	0.9900
N1—N2	1.358 (4)	C9—C10	1.518 (5)
N2—C4	1.337 (5)	C9—H9A	0.9900
N3—C4	1.378 (4)	C9—H9B	0.9900
N3—C5	1.459 (5)	C10—C11	1.509 (5)
N3—C8	1.469 (4)	C10—H10A	0.9900
N4—C9	1.462 (4)	C10—H10B	0.9900
N4—C7	1.465 (4)	C11—H11A	0.9900
N4—C6	1.466 (4)	C11—H11B	0.9900
C1—C2	1.392 (5)	C12—C17	1.389 (5)
C2—C3	1.357 (5)	C12—C13	1.391 (5)
C2—H2	0.9500	C13—C14	1.383 (5)
C3—C4	1.419 (5)	C13—H13	0.9500
C3—H3	0.9500	C14—C15	1.382 (5)
C5—C6	1.518 (5)	C14—H14	0.9500
C5—H5A	0.9900	C15—C16	1.383 (5)
C5—H5B	0.9900	C16—C17	1.392 (5)
C6—H6A	0.9900	C16—H16	0.9500
C6—H6B	0.9900	C17—H17	0.9500
			
C12—O1—C11	116.9 (3)	C7—C8—H8A	109.6
C1—N1—N2	118.6 (3)	N3—C8—H8B	109.6
C4—N2—N1	119.4 (3)	C7—C8—H8B	109.6
C4—N3—C5	118.2 (3)	H8A—C8—H8B	108.1
C4—N3—C8	119.4 (3)	N4—C9—C10	113.7 (3)
C5—N3—C8	111.7 (3)	N4—C9—H9A	108.8
C9—N4—C7	112.2 (3)	C10—C9—H9A	108.8
C9—N4—C6	108.8 (3)	N4—C9—H9B	108.8
C7—N4—C6	108.8 (3)	C10—C9—H9B	108.8
N1—C1—C2	125.2 (3)	H9A—C9—H9B	107.7
N1—C1—Cl1	114.9 (3)	C11—C10—C9	113.2 (3)
C2—C1—Cl1	119.9 (3)	C11—C10—H10A	108.9
C3—C2—C1	116.8 (3)	C9—C10—H10A	108.9
C3—C2—H2	121.6	C11—C10—H10B	108.9
C1—C2—H2	121.6	C9—C10—H10B	108.9
C2—C3—C4	117.6 (4)	H10A—C10—H10B	107.7
C2—C3—H3	121.2	O1—C11—C10	108.0 (3)
C4—C3—H3	121.2	O1—C11—H11A	110.1
N2—C4—N3	117.0 (3)	C10—C11—H11A	110.1
N2—C4—C3	122.4 (3)	O1—C11—H11B	110.1
N3—C4—C3	120.5 (3)	C10—C11—H11B	110.1
N3—C5—C6	109.8 (3)	H11A—C11—H11B	108.4
N3—C5—H5A	109.7	O1—C12—C17	124.1 (3)
C6—C5—H5A	109.7	O1—C12—C13	115.9 (3)
N3—C5—H5B	109.7	C17—C12—C13	120.0 (3)
C6—C5—H5B	109.7	C14—C13—C12	120.3 (3)
H5A—C5—H5B	108.2	C14—C13—H13	119.8
N4—C6—C5	112.1 (3)	C12—C13—H13	119.8
N4—C6—H6A	109.2	C15—C14—C13	119.3 (3)
C5—C6—H6A	109.2	C15—C14—H14	120.4
N4—C6—H6B	109.2	C13—C14—H14	120.4
C5—C6—H6B	109.2	C14—C15—C16	121.2 (3)
H6A—C6—H6B	107.9	C14—C15—Cl2	119.6 (3)
N4—C7—C8	111.3 (3)	C16—C15—Cl2	119.3 (3)
N4—C7—H7A	109.4	C15—C16—C17	119.5 (4)
C8—C7—H7A	109.4	C15—C16—H16	120.3
N4—C7—H7B	109.4	C17—C16—H16	120.3
C8—C7—H7B	109.4	C12—C17—C16	119.8 (3)
H7A—C7—H7B	108.0	C12—C17—H17	120.1
N3—C8—C7	110.4 (3)	C16—C17—H17	120.1
N3—C8—H8A	109.6		
			
C1—N1—N2—C4	−0.9 (5)	C4—N3—C8—C7	−160.2 (3)
N2—N1—C1—C2	2.5 (5)	C5—N3—C8—C7	56.0 (4)
N2—N1—C1—Cl1	−177.3 (2)	N4—C7—C8—N3	−57.3 (4)
N1—C1—C2—C3	−1.9 (5)	C7—N4—C9—C10	57.1 (4)
Cl1—C1—C2—C3	177.8 (2)	C6—N4—C9—C10	177.6 (3)
C1—C2—C3—C4	−0.2 (5)	N4—C9—C10—C11	172.0 (3)
N1—N2—C4—N3	−178.7 (3)	C12—O1—C11—C10	−175.1 (3)
N1—N2—C4—C3	−1.1 (5)	C9—C10—C11—O1	−71.7 (4)
C5—N3—C4—N2	0.0 (5)	C11—O1—C12—C17	−7.1 (5)
C8—N3—C4—N2	−141.5 (3)	C11—O1—C12—C13	173.0 (3)
C5—N3—C4—C3	−177.5 (3)	O1—C12—C13—C14	−178.8 (3)
C8—N3—C4—C3	41.0 (5)	C17—C12—C13—C14	1.3 (5)
C2—C3—C4—N2	1.6 (5)	C12—C13—C14—C15	−0.8 (5)
C2—C3—C4—N3	179.1 (3)	C13—C14—C15—C16	−0.5 (5)
C4—N3—C5—C6	160.3 (3)	C13—C14—C15—Cl2	179.6 (3)
C8—N3—C5—C6	−55.4 (4)	C14—C15—C16—C17	1.2 (5)
C9—N4—C6—C5	179.6 (3)	Cl2—C15—C16—C17	−178.9 (3)
C7—N4—C6—C5	−57.9 (4)	O1—C12—C17—C16	179.5 (3)
N3—C5—C6—N4	57.0 (4)	C13—C12—C17—C16	−0.6 (5)
C9—N4—C7—C8	178.2 (3)	C15—C16—C17—C12	−0.6 (5)
C6—N4—C7—C8	57.7 (3)		

### Hydrogen-bond geometry (Å, °)

**Table:**

*D*—H···*A*	*D*—H	H···*A*	*D*···*A*	*D*—H···*A*
C3—H3···N1^i^	0.95	2.53	3.247 (6)	133
C3—H3···N2^i^	0.95	2.50	3.427 (6)	164
C13—H13···O1^ii^	0.95	2.60	3.529 (5)	168

Symmetry codes: (i) *x*, *y*+1, *z*; (ii) −*x*+3/2, −*y*−1/2, −*z*+1.
